# N-alpha-acetylation of Huntingtin protein increases its propensity to aggregate

**DOI:** 10.1016/j.jbc.2021.101363

**Published:** 2021-10-31

**Authors:** Leah Gottlieb, Lin Guo, James Shorter, Ronen Marmorstein

**Affiliations:** 1 Department of Biochemistry and Biophysics, Perelman School of Medicine, University of Pennsylvania, Philadelphia, Pennsylvania, USA; 2 Abramson Family Cancer Research Institute, Perelman School of Medicine, University of Pennsylvania, Philadelphia, Pennsylvania, USA

**Keywords:** NatA, N-terminal acetylation, Huntington disease, Huntingtin, aggregation, post-translational modification (PTM), cotranslational modification, neurodegenerative disease, biophysics, (GS)_4_, four–Gly–Ser linker, 6xHis, 6xhistidine, Ac-HttQ25, N-terminally acetylated HttQ25, DLS, dynamic light scattering, DSS, disuccinimidyl suberate, EGS, ethylene glycol bis(succinimidyl succinate, HD, Huntington’s disease, hNatA, humanNatA, Hsf1, heat-shock factor 1, Htt, Huntingtin, HttQ25, 25 polyQ, HttQ44, 44 polyQ, N17, 17 N-terminal Htt residues, NAT, N-terminal acetyltransferase, polyQ, poly-glutamine, SUMO, small ubiquitin-like modifier, *t*_*50*_, aggregation half-time, TEM, transmission electron microscopy, TEV, tobacco etch virus, αSyn, α-synuclein

## Abstract

Huntington’s disease (HD) is a neurodegenerative disorder caused by a poly-CAG expansion in the first exon of the *HTT* gene, resulting in an extended poly-glutamine tract in the N-terminal domain of the Huntingtin (Htt) protein product. Proteolytic fragments of the poly-glutamine–containing N-terminal domain form intranuclear aggregates that are correlated with HD. Post-translational modification of Htt has been shown to alter its function and aggregation properties. However, the effect of N-terminal Htt acetylation has not yet been considered. Here, we developed a bacterial system to produce unmodified or N-terminally acetylated and aggregation-inducible Htt protein. We used this system together with biochemical, biophysical, and imaging studies to confirm that the Htt N-terminus is an *in vitro* substrate for the NatA N-terminal acetyltransferase and show that N-terminal acetylation promotes aggregation. These studies represent the first link between N-terminal acetylation and the promotion of a neurodegenerative disease and implicates NatA-mediated Htt acetylation as a new potential therapeutic target in HD.

Huntington’s disease (HD) is a fatal autosomal neurodegenerative disease caused by a poly-CAG expansion in the first exon of the *HTT* gene. Upon onset, patients with HD progressively develop a range of symptoms, including dementia, involuntary movements, and psychiatric disturbances, which is accompanied by neuronal degeneration in the striatum and cortex. Death typically occurs ∼10 to 25 years after diagnosis (reviewed in (1)). The CAG expansion codes for a poly-glutamine (polyQ) tract in the N-terminal domain of the ∼3144-residue Huntingtin (Htt) protein product. Htt polyQ tract expansions longer than 35 are penetrant with a direct relationship between expansion length and both disease severity and age of onset. Moreover, the length of the Htt polyQ expansion is proportionally correlated with its increased propensity to aggregate and form both fibrils and intranuclear inclusion bodies (2, 3, 4). Interestingly, there have been reports of patients with polyQ tracts featuring lengths between 29 and 34 having similar phenotypes to HD, suggesting that, in addition to polyQ length dependency, additional mechanisms may influence HD pathogenesis (5, 6, 7, 8, 9, 10, 11). However, the diagnoses remain contested (12, 13). Moreover, pathogenic polyQ expansions (∼40–64 repeats) in Htt have also been found in rare cases of amyotrophic lateral sclerosis and frontotemporal dementia that do not display any features of HD (14).

The full-length Htt protein undergoes proteolysis, leading to the production of a pathogenic N-terminal fragment containing the polyQ tract (15, 16). Htt proteolytic fragments have been found in both the cytosol and the nucleus and, particularly, in the characteristic neuronal intranuclear inclusion bodies (17, 18). The resulting diseased truncation is sufficient for the induction of aggregation (19) as well as production of the HD phenotype in model systems (20, 21). Therefore, numerous studies have been conducted using a minimal N-terminal fragment construct containing the 17-residue N-terminal domain (N17), a polyQ repeat (Q_n_), and, in some cases, the C-terminal polyproline sequence to shed light on the mechanisms governing Htt aggregation. N-terminal and C-terminal flanking sequences of Htt have recently emerged as regulators of Htt aggregation and function (22, 23, 24, 25, 26, 27, 28, 29, 30, 31, 32, 33). While the polyproline domain appears to confer protective qualities against mutant Htt aggregation, the N-terminal sequence (MATLEK-) has been found to contribute to aggregation (27, 32, 34). Moreover, the Htt N-terminal domain serves as a primary binding site for interaction with some protein chaperones that are capable of suppressing Htt aggregation (35, 36, 37).

Post-translational modification of the N-terminal domain alters Htt function and aggregation. Modifications studied to date include lysine acetylation (38, 39), palmitoylation (40), phosphorylation (41), SUMOylation (42), and ubiquitination (43, 44, 45, 46, 47). However, the influence of N-terminal processing and, particularly, N-terminal acetylation has not yet been considered. Instead, numerous studies have either incorporated N-terminal tags to solubilize the recombinant Htt species or included an N-terminal methionine in the preparation of synthetic Htt peptides.

With ∼80% of the human protein being N-terminally acetylated, this co- and post-translational modification has broad implications in the regulation of cellular processes and human development and disease (48, 49). The N-alpha-acetyl mark has been demonstrated to influence protein–protein interactions, protein–membrane interactions, and localization, among other functions (reviewed in (50)). The metazoan-conserved N-terminal acetyltransferases (NATs) are responsible for the transfer of the acetyl mark from acetyl coenzyme A to the target peptide N-terminus. To date, seven NATs (NatA-F and H) have been identified in humans, each consisting of a catalytic subunit (NAA10-NAA60 and NAA80, respectively) and potentially up to two auxiliary subunits. These enzymes specifically target substrates based on the first two and, in some cases, additional residues in the N-terminus. In the case of NatA, the N-termini must have been processed by the methionine aminopeptidase such that excision exposes a small residue. A previous study demonstrated that knockdown of the NAA10 catalytic subunit of the NatA complex or its regulatory binding partner, HYPK, resulted in an increase in Htt aggregation (51), thus implicating a role for NatA-mediated N-terminal Htt acetylation in its aggregation properties.

The N-terminal sequence of Htt appears to play a role in its aggregation, but the exact role is still being investigated (26, 52). Multiple studies have described the acceleration of Htt aggregation through N17-promoted Htt Exon1 oligomerization followed by polyQ-promoted aggregation (35, 53, 54, 55). Interestingly, the N-terminus of Htt corresponds to a canonical NatA complex substrate, ATLEK-. More recently, it was confirmed both WT and mutant Htt produced in HEK293 cells are N-terminally processed to yield an acetylated N-terminus: Ac-ATLEK-, which purified as a dimer and was able to form higher-order oligomers (56). In addition, the mouse brain has been reported to have N-terminally acetylated Htt protein (57).

Based on these earlier studies, we set out to characterize the effect of NatA-mediated acetylation on Htt aggregation and stability. Thus, we developed a bacterial system to produce unmodified or N-terminally acetylated Htt N-terminus and used this system to confirm that the Htt N-terminus is an *in vitro* substrate for NatA. Although N-terminally acetylated synthetic N-terminal Htt peptides did not appear to significantly alter the random coil character, the acetyl mark promoted aggregation of a polyQ-containing N-terminal fragment. These studies represent the first model where N-terminal acetylation promotes Htt protein aggregation, which has implications for adverse roles in neurodegenerative disease and targeting NatA in HD.

## Results

### Development of Htt polyQ N-terminal fragment constructs for *in vitro* studies

**Figure 1:**
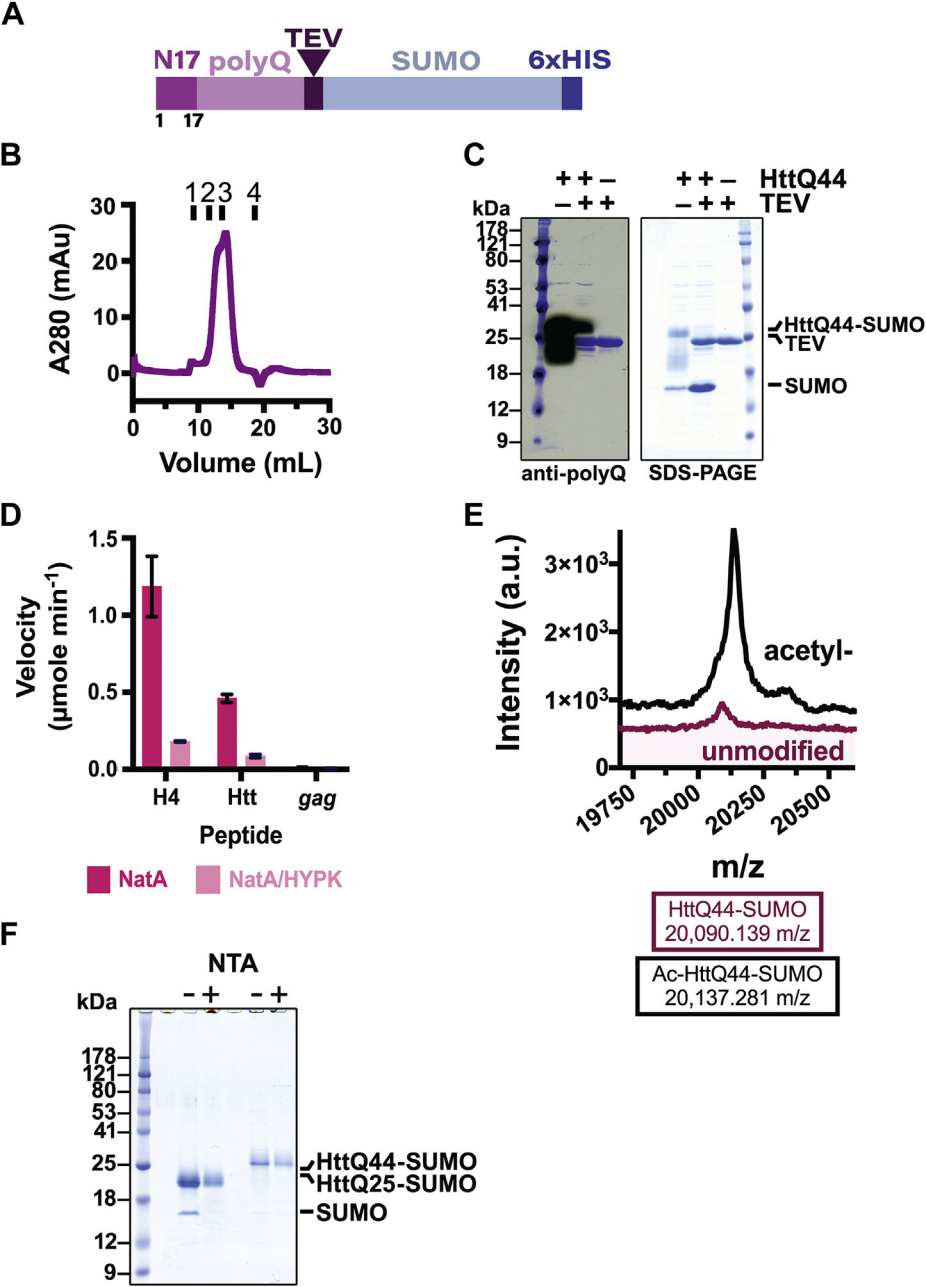
**Production of unmodified and N-terminally acetylated Htt N-terminal fragment recombinant proteins**. *A*, schematic of recombinant Htt protein constructs. The Htt fragment encodes for the 17 N-terminal residues (N17, *dark pink*) and a poly-glutamine tract (polyQ, *light pink*) and a tobacco etch virus (TEV)-cleavage site (*dark purple*) that is C-terminal to the SUMO and 6xHIS tags (*cyan* and *dark blue*, respectively). Constructs contain a polyQ tract with either 25 or 44 repeats. *B*, Superdex 75 gel-filtration chromatogram of monomeric C-terminally SUMO-tagged HttQ44 (HttQ44-SUMO) with retention volumes of protein standards indicated (1: 164 kDa; 2: 44 kDa; 3: 17 kDa; 4: 1.37 kDa). *C*, TEV cleavage of gel filtration–purified HttQ44-SUMO–performed overnight at ambient temperature–visualized by Western blot using an anti-polyQ (MW1, *left*) primary antibody and SDS-PAGE stained with Coomassie Brilliant Blue (*right*). *D*, bar graph showing the N-terminal acetyltransferase activity of NatA (*magenta*) and NatA/HYPK (*light pink*) against peptides corresponding to the *in vitro* NatA substrate, histone H4, Htt, and the *S. cerevisiae* NatC substrate, *gag*. Error bars correspond to the SD and n = 3 technical replicates. *E*, MALDI spectra of unmodified HttQ44-SUMO (*magenta*) and Ac-HttQ44-SUMO (*black*). *F*, SDS-PAGE of all uncleaved purified Htt proteins: unmodified (−) and N-terminally acetylated (+) HttQ25-SUMO and HttQ44-SUMO. Ac-HttQ25, N-terminally acetylated HttQ25; Htt, Huntingtin; HttQ44, 44 polyQ; SUMO, small ubiquitin-like modifier.

To directly interrogate the involvement of the Htt N-terminus in Htt aggregation, we developed an aggregation-inducible recombinant N-terminal Htt protein system (Fig. 1*A*). The Htt fragment encodes the first 17 residues (N17), followed by either a 25-polyQ (“HttQ25”) or an expanded 44-polyQ (“HttQ44”) tract and a set of C-terminal tags: a tobacco etch virus (TEV)-cleavable small ubiquitin-like modifier (SUMO) for improving Htt solubility and a 6xhistidine (6xHis) tag for affinity purification (2, 3, 4) (Fig. S1). We then expressed the protein constructs in *Escherichia coli* and purified them to homogeneity using gel filtration chromatography as a polishing step (Fig. 1*B*). Using Edman degradation sequencing, we confirmed that the initiator methionine had been excised from the N-terminus of the HttQ44 construct, leaving the Ala2 residue exposed (data not shown). Furthermore, we confirmed that TEV protease can successfully cleave the C-terminal SUMO tag from the HttQ44 construct (Fig. 1*C*).

The Htt N-terminal sequence corresponds to a predicted NatA complex substrate. To evaluate whether Htt may be a substrate of the human NatA complex, we used an *in vitro* radioactive NAT assay to assess human NatA and NatA–HYPK complex activity toward a series of synthetic peptides, including one with the first 7 residues of Htt after methionine excision (ATLEKLM) (Fig. 1*D*). The *in vitro* assay revealed that both the human NatA and NatA–HYPK complexes can acetylate the N-terminus of an Htt peptide. Consistent with previous data, the measured velocity for the human NatA–HYPK complex toward both the Htt peptide and the *in vitro* substrate histone H4 peptide was demonstrably lower than that of the human NatA complex because of the intrinsic human NatA-specific inhibitor activity of the regulatory binding partner, HYPK (58). Based on these data and previous studies performed with N-terminally acetylated α-synuclein (αSyn) produced by its co-expression with *Schizosaccharomyces* *pombe* NatB in *E. coli* (57, 58), we coexpressed HttQ44 with *S. pombe* NatA and purified the N-terminally acetylated HttQ44 to homogeneity. Using MALDI MS, we confirmed that HttQ44 is indeed N-terminally acetylated (Fig. 1*E*). Finally, based on the work conducted above, we also expressed and purified recombinant HttQ25 and the N-terminally acetylated HttQ25 (Ac-HttQ25) for parallel aggregation studies (Fig. 1*F*).

### N-terminal acetylation increases Htt aggregation propensity

**Figure 2:**
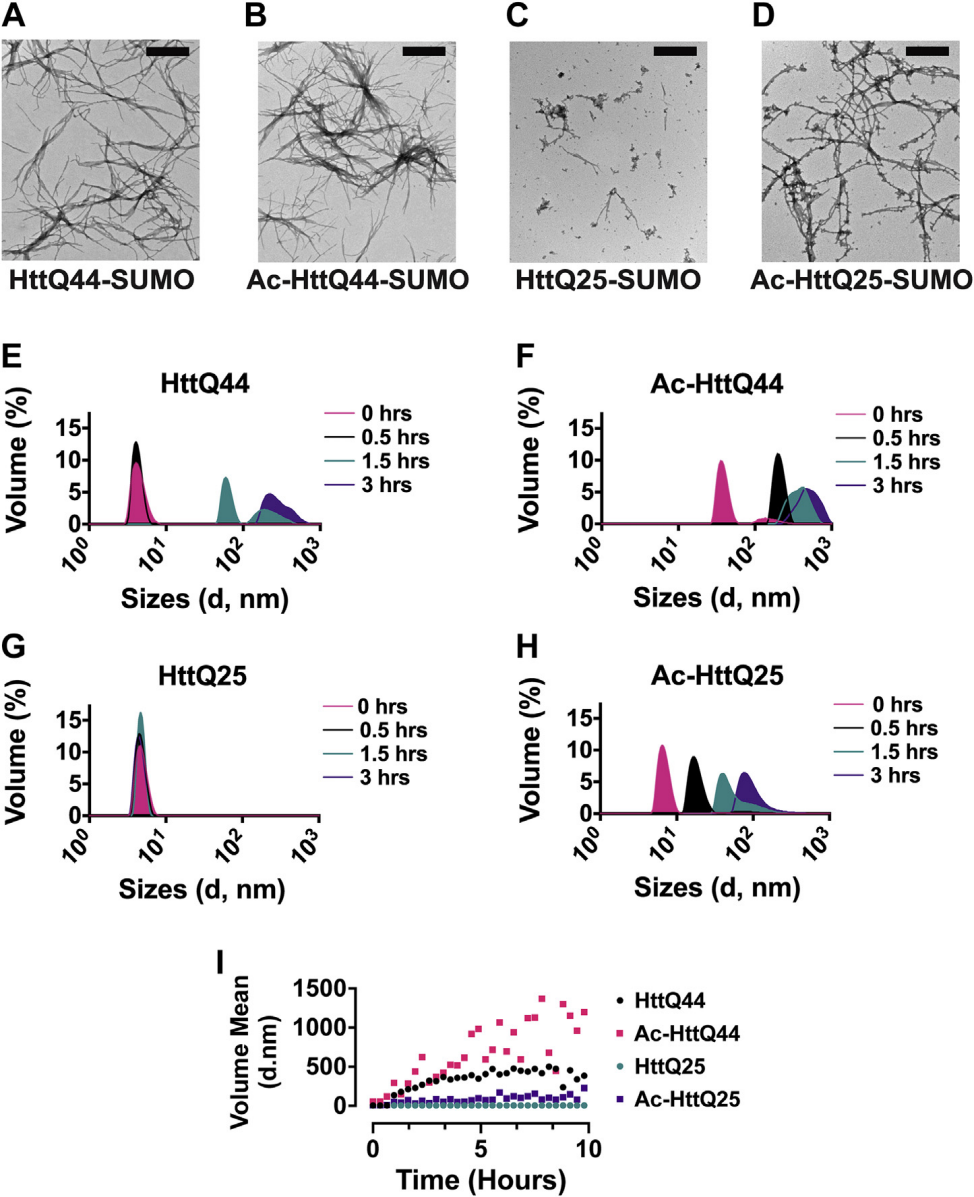
**N-terminal acetylation promotes Htt fibrilization.***A*–*D*, representative electron micrograph images containing SUMO-tagged Htt proteins (10 μM), (*A*) HttQ44-SUMO; (*B*) Ac-HttQ44-SUMO; (*C*) HttQ25-SUMO; and (*D*) Ac-HttQ25-SUMO, sampled after overnight incubation at ambient temperatures. The scale bars are 400 nm. *E*–*I*, DLS time course monitoring changes in Htt protein (20.9 μM) particle sizes induced by TEV cleavage at t = 0 h, 0.5 h, 1.5 h, and 3 h at 37 °C in the sizing buffer where t = 0 refers to the first measurement after introduction of TEV. *E*, HttQ44; (*F*) Ac-HttQ44; (*G*) HttQ25; and (*H*) Ac-HttQ25. DLS assays were performed in triplicate as technical replicates. Representative traces are shown. *I*, corresponding scatterplot of change in the volume mean over time. Ac-HttQ25, N-terminally acetylated HttQ25; DLS, dynamic light scattering; HttQ44, 44 polyQ; SUMO, small ubiquitin-like modifier; TEV, tobacco etch virus.

To evaluate the effects of NatA-mediated N-terminal acetylation on Htt aggregation, we first used transmission electron microscopy (TEM) (Figs. 2, *A*–*D* and S2) and incubated the four Htt variants (10 μM: HttQ44-SUMO and Ac-HttQ44-SUMO; 8 μM: HttQ25-SUMO and Ac-HttQ25-SUMO) overnight in parallel at room temperature (RT). Both the unmodified and N-terminally acetylated HttQ44 appeared to produce fibrils (Fig. 2, *A* and *B*). Unexpectedly, we found that Ac-HttQ25 also produced fibrils. Consistent with previous studies, the unmodified HttQ25 exhibited minimal fibril formation (Fig. 2, *C* and *D*).

We then monitored the changes in the Htt particle size during the earlier stages of Htt aggregation using dynamic light scattering (DLS) (Figs. 2, *E*–*I* and S3). Similar to previous studies, the signal-to-noise ratio at lower concentrations was insufficient (59), causing us to conduct the DLS assays using a higher concentration of Htt protein (∼20 μM). Consistent with results of our TEM studies, we did not observe the development of significantly larger species when monitoring the unmodified HttQ25 over the duration of the experiment (Fig. 2*G*). Although we found that Ac-HttQ25 was initially the same diameter as HttQ25 at t = 0, it quickly grew over the 3-h time span (Fig. 2*H*). With respect to HttQ44 and Ac-HttQ44, the unmodified HttQ44 initially appeared to maintain its size distribution over the first 30 min and then proceeded to grow (Fig. 2*E*). The initial size of Ac-HttQ44 was significantly larger than HttQ44 and proceeded to grow into larger size species at a faster rate (Fig. 2, *F* and *I*), together indicating that N-terminal acetylation promotes the formation of an aggregation-prone nucleated species.

**Figure 3:**
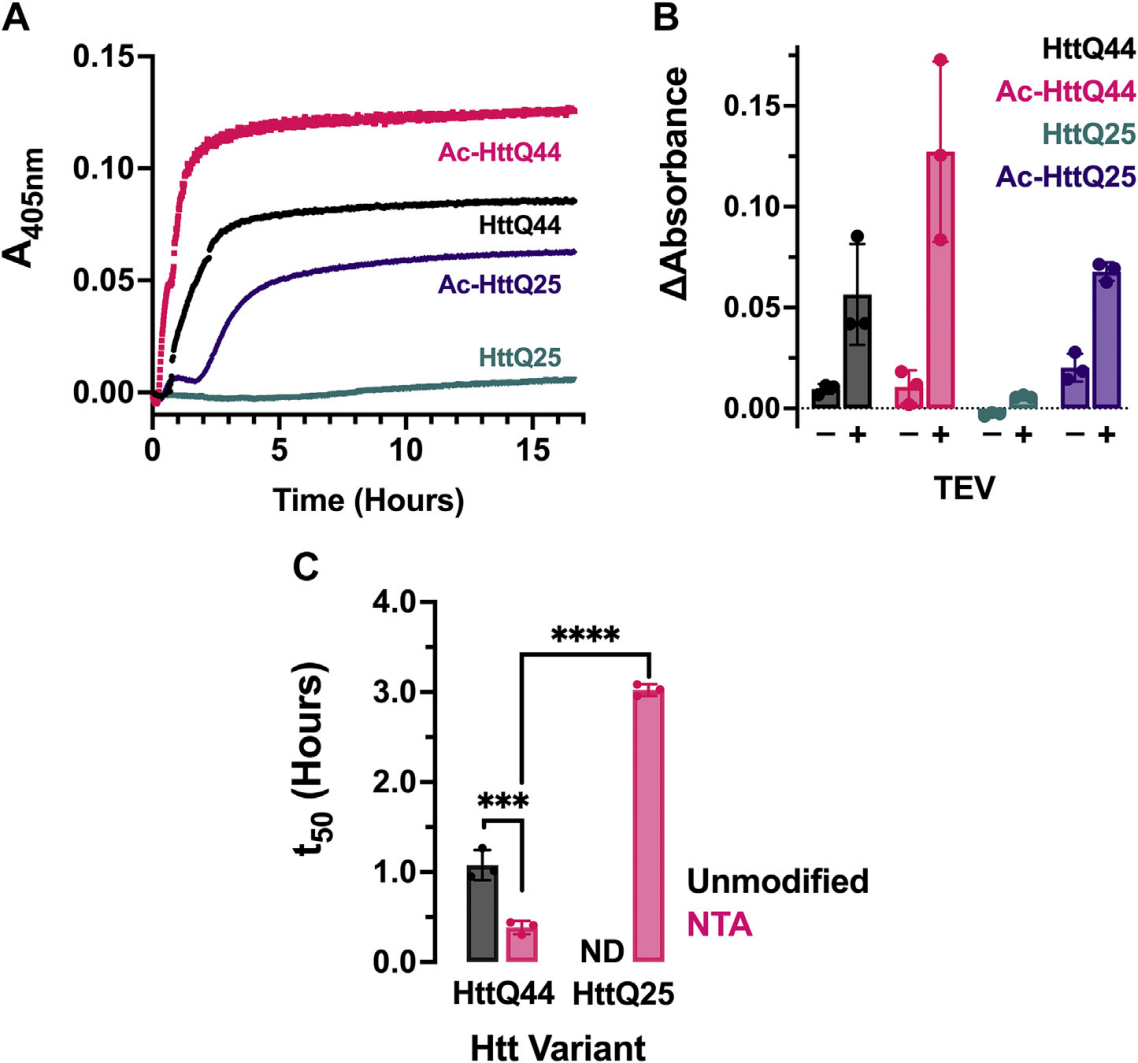
**N-terminal acetylation influences Htt aggregation kinetics.***A*, Htt (10 μM) aggregation monitored by turbidity without agitation at an absorbance of 405 nm with all four TEV-cleaved constructs overlayed. Unmodified HttQ44 (*black*), N-terminally acetylated HttQ44 (Ac-HttQ44, *pink*), unmodified HttQ25 (*teal*), and N-terminally acetylated HttQ25 (*purple*, Ac-HttQ25) were incubated overnight with TEV protease at 37 °C. *B*, bar graph of change in absorbance at 405 nm for untreated (−) and TEV-treated (+) Htt proteins: unmodified HttQ44 (*black*), N-terminally acetylated HttQ44 (Ac-HttQ44, *pink*), unmodified HttQ25 (*teal*), and N-terminally acetylated HttQ25 (*purple*, Ac-HttQ25). *C*, bar graphs depicting *t*_*50*_ – calculated using a nonlinear regression fit with a three-parameter dose response curve. Error bars represent the SD. Significance calculated using a two-way ANOVA and correction for multiple comparison using a Tukey’s multiple comparisons test, where alpha = 0.05. Significance designated as ∗∗∗∗ *p* ≤ 0.00001 and ∗∗∗ *p* = 0.0003; n = 3 technical replicates. Htt, Huntingtin; HttQ25, 25 polyQ; HttQ44, 44 polyQ; TEV, tobacco etch virus.

To quantify the differences in aggregation half-times (*t*_*50*_) between the unmodified and N-terminally acetylated Htt species, we monitored their aggregation using turbidity assays. After addition of TEV protease, we monitored the turbidity of Htt proteins (10 μM) at 37 °C without agitation over ∼16.5 h using a wavelength of 405 nm (Figs. 3 and S3).

Consistent with previous studies, the aggregation half-time, *t*_*50*_, (Fig. 3*C*, top) inversely related to the length of the polyQ repeat (3, 27, 36, 60, 61, 62, 63). Notably, N-terminal acetylation reduced the half-time by ∼47% for HttQ44 (from 1.217 ± 0.174 h to 0.649 ± 0.124 h). However, we were unable to determine the half-time for the unmodified HttQ25 because the HttQ25 aggregation did not plateau. We were able to determine the half-time of Ac-HttQ25, which was ∼79% longer than the N-terminally acetylated HttQ44.

### N-terminal acetylation does not significantly alter Htt peptide secondary structure

**Figure 4:**
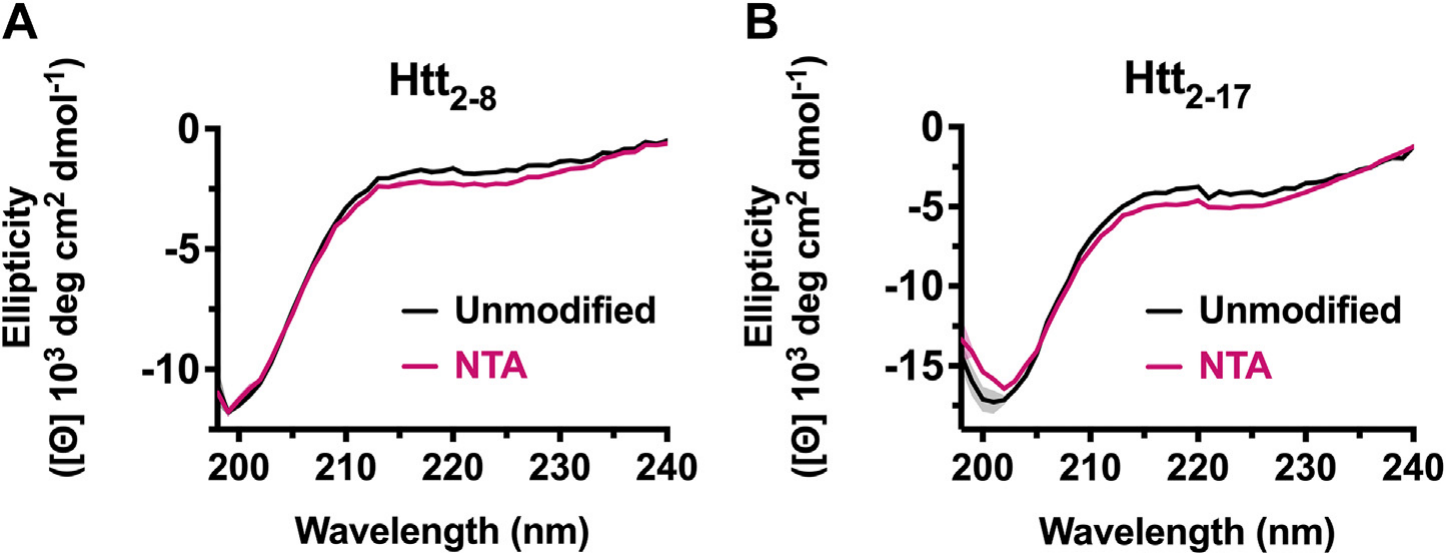
**N-terminal acetylation marginally alters the structure of Htt N**-**terminus.** CD spectra of Htt N-terminal peptides varying in length from (*A*) 7 residues (Htt_2–8_) to (*B*) 16 residues (Htt_2–17_) either without modification (*black line*; 200 and 36 μM, respectively) or with N-terminal acetylation (NTA), (*A*) Ac-Htt_2–8_ and (*B*) Ac-Htt_2–17_ (*magenta line*; 245 μM and 68 μM, respectively) acquired at 37 °C. *Curves* represent the average of three separate replicates after buffer scan correction; error bands represent the SD. Htt, Huntingtin.

Previous studies using Htt proteins with the methionine intact have implicated the N-terminal flanking domain in promoting Htt aggregation through its α-helical structure (35, 36, 60, 61, 62, 64). To evaluate the effect of N-terminal acetylation on the secondary structure of the methionine-excised Htt N-terminus, we used CD spectroscopy. We evaluated synthetic Htt peptides corresponding to the methionine-excised Htt protein product either with an unmodified or N-terminally acetylated N-terminus. Peptides differed in length; sequences were either seven (7mer; Htt_2–8_ or Ac-Htt_2–8_) or 16 residues long (16mer; Htt_2–17_ or Ac-Htt_2–17_). Here, we found that, consistent with previous studies (27, 30, 61, 63), all of the unmodified peptides, Htt_2–8_ and Htt_2–17_, featured random coil characteristics (Fig. 4, *A* and *B*). Both N-terminal peptides appeared to result in a small increase in the signal at 220 nm and decrease in 208 nm, which was more pronounced with the longer peptide and consistent with greater α-helix content. However, the observed changes were relatively modest, suggesting that the N-terminal acetylation of these Htt peptides does not significantly alter its structure. Interestingly, these data using synthetic peptides and the magnitude of change observed resemble studies performed using full-length αSyn protein, which have demonstrated that N-terminal acetylation of αSyn also marginally increases its N-terminal α-helical propensity, particularly in the presence of a membrane (57, 65).

### N-terminal acetylation alters Htt oligomerization

**Figure 5:**
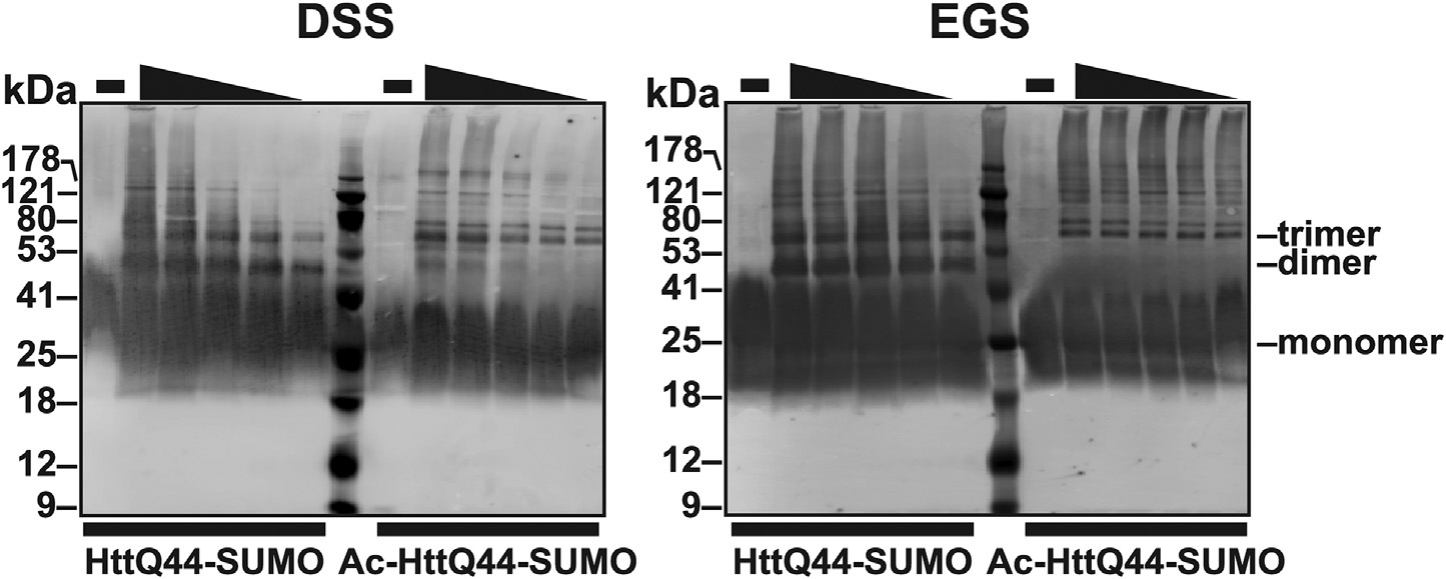
**Chemical crosslinking studies reveal that N-terminal acetylation alters Htt aggregation pattern.** Western blot visualization of unmodified and N-terminally acetylated HttQ44-SUMO (23.7 μM) alone (−) and exposed to decreasing concentrations (75 mM, 37.5 mM, 18.8 mM, 9.4 mM, and 4.7 mM) of the crosslinker: disuccinimidyl suberate (DSS, *left*) or ethylene glycol bis(succinimidyl succinate (EGS, *right*). Reactions were performed at room temperature and quenched using 1 M Tris, pH 7.5. Reactions were boiled with the SDS-PAGE loading buffer, loaded onto a 15% SDS-PAGE gel, transferred to PVDF, and probed using a mouse-derived monoclonal anti-polyQ primary antibody and IRDye 680RD Goat anti-mouse secondary antibody for LI-COR image acquisition. Proposed oligomerization states are indicated to the *right* (monomer, dimer, and trimer). Htt, Huntingtin; polyQ, poly-glutamine; SUMO, small ubiquitin-like modifier.

Owing to the striking differences in aggregation half times between the unmodified and N-terminally acetylated HttQ44, we sought to evaluate the effects of N-terminal acetylation on HttQ44 oligomerization. Therefore, we exposed either unmodified or N-terminally acetylated HttQ44 (23.7 μM) to one of two chemical crosslinkers with varying spacer arm lengths at varying concentrations (0, 4.7, 9.4, 18.8, 37.5, and 75 mM): disuccinimidyl suberate (DSS, 11.4 Å spacer) or ethylene glycol bis(succinimidyl succinate (EGS, 16.1 Å spacer). After a 30-min incubation at RT, reactions were quenched and loaded onto an SDS-PAGE gel for visualization by Western blot using an anti-polyQ antibody (Fig. 5).

The resulting Western blot revealed that both the unmodified and N-terminally acetylated HttQ44 samples contained a mixture of oligomerization states. While the unmodified HttQ44 appeared to contain dimers, trimers, and other higher order oligomers, Ac-HttQ44 showed very little evidence of a dimer and, instead, appeared to consist primarily of trimers and other higher order oligomers.

Together, these data indicate that N-terminal acetylation alters Htt to favor trimer formation in the early stages of aggregation. Ultimately, these differences enhance Ac-HttQ44 and Ac-HttQ25 fibrilization compared with unmodified HttQ44 and unmodified HttQ25 fibrilization.

## Discussion

While previous studies have reported on the role of post-translational modifications in HD, the effects of the co-translational N-terminal acetyl mark on Htt aggregation has not been described. With an Htt N-terminal cognate sequence containing ATLEK−, Htt corresponds to a putative substrate for the NatA, which is responsible for N-terminal acetylation of most human proteins. Indeed, it has been reported that Htt is N-terminally acetylated in the mouse brain (57) and when produced in both HEK293 and insect cells (*Spodoptera* *frugiperda*, Sf9) cells (56). These findings led us to evaluate a possible role for NatA complex–mediated Htt N-terminal acetylation in HD. To do so, we used biochemically pure reagents to confirm that NatA can acetylate the N-terminus of Htt, developed a bacterial system to produce unmodified and N-terminally acetylated Htt, and used these systems to evaluate the biophysical and aggregation properties of penetrant (HttQ44) and nonpenetrant (HttQ25) Htt constructs.

**Figure 6:**
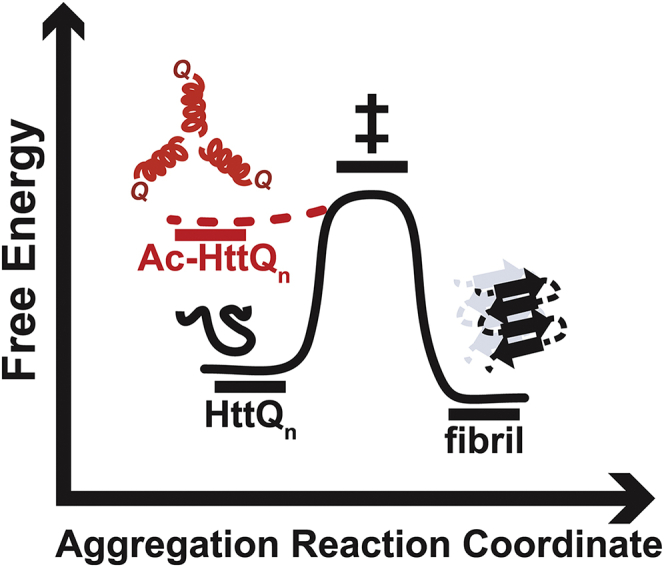
**N-terminal acetylation drives Htt fibrillization.** Cartoon representation of our proposed model using an aggregation-free energy diagram. The diagram describes the destabilizing effect of N-terminal acetylation (Ac-, *red dotted lines*) on Htt aggregation. Htt, Huntingtin.

Our studies demonstrate that Htt N-terminal acetylation promotes Htt aggregation. This stimulation is likely mediated *via* the destabilized, oligomerization-promoting N-terminal amphipathic α-helix, as previously reported (22, 28, 30, 66). We propose that destabilization of Htt by N-terminal acetylation helps drive aggregation by lowering the energy barrier for fibrillization (Fig. 6). In future studies, it would be important to quantify the extent of N-terminal Htt acetylation in cells. Our attempts to do this using immunoprecipitation and LC-MS/MS analyses of either endogenous or overexpressed Htt in human cells were unsuccessful, likely because of the inability to quantitatively identify N-terminal Htt peptides after proteolytic digestion, regardless of the N-terminal acetylation status.

Owing to its intrinsic NatA-inhibitory activity, the NatA binding partner, HYPK, may play a role in limiting NatA complex co-translational activity and, consequently, Htt aggregation. Consistent with this possibility, HYPK knockdown leads to an increase in Htt aggregation in tissue culture models (51, 67, 68, 69). Moreover, HYPK is reported to be downregulated in both cell and animal models of HD (70), likely because of a reduction in the occupancy of the *HYPK* promoter by an impaired heat-shock factor 1 (Hsf1) transcription factor (67, 71). Indeed, Hsf1 is degraded in HD models as well as depleted in both differentiated human inducible pluripotent stem cells and both the striatum and cortex of patients with HD (72).

Our study is not the first account describing the influence of N-terminal acetylation in altering protein aggregation. In yeast, NatA modifies the yeast prion protein Sup35. Loss of NatA activity diminishes the yeast prion, [*PSI*^+^], phenotype by reducing Sup35 aggregation and causing an Hsf1-induced heat shock response and, consequently, chaperone-mediated prion clearance (73, 74). In contrast, human NatB-mediated N-terminal acetylation of αSyn, the major protein involved in Parkinson’s disease, plays a more nuanced role in its aggregation. Although N-terminal acetylation of WT αSyn reduces its aggregation, N-terminal acetylation of αSyn containing some familial Parkinson’s disease mutations can enhance its aggregation kinetics (58, 75). However, it has also been observed that N-terminal acetylation promotes intracellular αSyn aggregation in primary neurons. Furthermore, the N-terminal acetylation of αSyn appears to play a role in facilitating its ability to interact with membranes and binding to N-linked glycans (58, 65, 66, 66, 75, 76, 77, 78). Therefore, the N-terminal acetylation of proteins can have different consequences in protein aggregation and disease.

Earlier studies, including a yeast two-hybrid assay, described a direct interaction between Htt and HYPK (37, 79, 80, 81). However, a dissociation constant for the interaction between Htt and HYPK has not been reported (82). Supporting the possibility that Htt does not interact directly with HYPK, Arnesen *et al.* demonstrated that Htt does not colocalize with HYPK in cells (51). Similarly, we were unable to detect a direct interaction between HYPK and Htt *in vitro* (Fig. S5). As a result, we propose that Htt is an *in vivo* substrate of the NatA and NatA–HYPK complexes, where the nascent Htt N-terminus comes in proximity of HYPK during translation. Indeed, yeast two-hybrid assays are prone to false positive results (83) and have only been able to detect stable substrate–enzyme interactions (84). In fact, in the same study, Arnesen *et al.* found that either NAA10 or HYPK knockdown only resulted in an increase in EGFP-tagged HttQ74 aggregation when the N-terminus remained untagged and the EGFP tag was engineered onto the C-terminus, consistent with a role for N17 N-terminal acetylation in promoting Htt aggregation.

Taken together, the data presented here suggest that N-terminal Htt acetylation promotes its aggregation properties and thus likely plays a stimulatory role in HD pathogenesis. These studies represent another link between N-terminal acetylation and the promotion of a neurodegenerative disease and points to NatA as a potential therapeutic target in HD.

## Experimental procedures

### Construction of *E. coli* expression vectors

Sequence encoding for the exon1 of the human Htt protein, which consists of the N-terminal 17 residues (MATLEKLMKAFESLKSF) and a 44-polyQ repeat, was engineered into the pET DUET vector and a pRSF vector both with a TEV protease-cleavable C-terminal SUMO tag, followed by a 6xHis tag. A similar construct containing a 25-polyQ tract was only engineered into the pRSF vector. The TEV cleavage site and SUMO-tag are bridged by a four–Gly–Ser linker [(GS)_4_]. The full-length *S. pombe* (*Sp*)Naa15p (729 residues) was engineered into MCSI of a pET DUET vector containing a TEV protease-cleavable 6XHis tag with MCSII containing Naa10p (1–156) to co-express the heterodimeric *Sp*NatA complex with Htt recombinant constructs (85). Genes encoding the HttQ25 and HttQ44 constructs described above were synthesized by Bio Basic and subcloned into the aforementioned vectors using NdeI and XhoI.

### Expression and purification of unmodified Htt proteins

HttQ25-(GS)_4_-SUMO-6xHis (HttQ25-SUMO) and HttQ44-(GS)_4_-SUMO-6xHis (HttQ44-SUMO) were both expressed using BL21(DE3) pLysS cells (Millipore), which were grown in LB media (Millipore) at 37 °C to an absorbance (A_600__nm_) of ∼0.5 to 0.6 and induced by addition of 0.5 mM IPTG at 16 °C for 16 h. All subsequent purification steps were carried out at 4 °C. Cells were isolated by centrifugation, lysed by sonication in the lysis buffer containing 25 mM Tris, pH 8.0, 150 M NaCl, 10 mM β-ME, and 10 mg m^l−1^ PMSF and DNAse. The lysate was clarified by centrifugation and passed over nickel resin, which was subsequently washed with >20 CV of the lysis buffer supplemented with 25 mM imidazole. The protein was eluted in the lysis buffer supplemented with 200 mM imidazole by batch elution. The 6xHis tag was cleaved overnight by addition of 6xHis-tagged Ulp1-protease during dialysis into the dialysis buffer containing 25 mM Tris, pH 8.0, 150 mM NaCl, and 10 mM β-ME. This solution was passed through an additional nickel column to remove Ulp1-protease as well as any uncut Htt and SUMO protein. This solution was loaded onto a 5-ml HiTrap Q ion exchange column. The protein was eluted in the same buffer with a salt gradient (150–750 mM NaCl) over the course of 18 CV (0–80% buffer B gradient). Peak fractions were pooled and concentrated (10-kDa concentrator) and loaded onto a Superdex 75 gel filtration column (GE Healthcare) in a buffer containing 25 mM Hepes, pH 7.0, 200 mM NaCl, and 1 mM TCEP. This protein was concentrated to 1 mg m^l−1^ as measured by A_280__nm_ as measured by a Nanodrop until use. Before each study, samples were thawed and spin-filtered to remove aggregates.

### Expression and purification of N-terminally acetylated Htt proteins

Similar to studies performed previously using coexpression of the *S. pombe* NatB with αSyn in *E. coli* to produce an N-terminally acetylated αSyn (57, 58, 65), both HttQ25-SUMO-6xHis and HttQ44_4_-SUMO-6xHis were co-expressed by co-transformation of BL21(DE3) pLysS cells (Millipore) with the *Sp*NatA plasmid. Cotransformed cells were grown in LB media (Millipore) at 37 °C to an A_600__nm_ of ∼0.5 to 0.6 and induced by addition of 0.5 mM IPTG at 16 °C for 16 h. All subsequent purification steps were carried out at 4 °C and are identical to the protocol implemented for the unmodified Htt proteins.

### Western blot analysis

SUMO-tag cleavage was performed overnight at RT such that the recombinant Htt was at a final concentration of 1.2 mg m^l−1^ and TEV was at a final concentration of 0.24 mg m^l−1^ in the sizing buffer. The reaction was quenched using 2X SDS loading dye followed by boiling. 15 μl of sample was loaded onto a 15% acrylamide gel analyzed by Western blot, using a 1:1000 dilution of a mouse-derived anti-polyQ monoclonal antibody (Sigma-Aldrich, cat # MABN2427) as the primary antibody in 5% nonfat milk and a 1:10,000 dilution of the secondary antibody, sheep-derived HRP-linked whole Ab ECL Mouse IgG (GE Healthcare, cat # 45-000-692), in 5% nonfat milk followed by a 30-s exposure.

### Edman degradation

A 50 μM sample of purified HttQ44_4_-SUMO-6xHis in the sizing buffer was submitted to the Molecular Structure Facility at the University of California, Davis Genome Center for Edman Sequencing. The recombinant Htt N-terminus was subjected to sequencing using five cycles of analysis.

### Radioactive acetylation of the Htt peptide

Human NatA (hNatA) acetyltransferase assays using hNatA and hNatA–HYPK protein prepared as described previously (86). The reactions were also set up as described previously where reactions were incubated with 10 nM of 6xHis-tagged hNatA in a 30-μl reaction volume containing 50 μM each of substrate peptide and [^14^C]acetyl-CoA (4 mCi/mmol; PerkinElmer Life Sciences) for 12 min at 25 °C. The substrate peptides (Genscript, described below) used in the assay corresponded to one of three peptides: the first 7 residues of Htt followed by a positively charged poly-arginine tag for electrostatic capture by the phosphocellulose papers used in the assays, the first 19 residues of human H4, or a control peptide with the first 7 residues of a non-NatA substrate (*gag*, a NatC substrate) followed by the same poly-arginine tag. The human histone H4 was selected to be consistent with previous studies performed with the hNatA and hNatA–HYPK complexes. Reactions lacking either peptide or enzyme were performed to measure background acetylation and confirm that any potential signal arising from chemical acetylation was negligible.

To quench the reaction, 20 μl of the reaction mixture was added to a negatively charged P81 phosphocellulose square (EMD Millipore), which was immediately placed in the wash buffer (10 mM Hepes, pH 7.5). The paper squares containing the captured peptides were washed for 5 min a total of 3 times to remove unreacted acetyl-CoA. The papers were air-dried after being briefly placed in acetone and then added to 4 ml of the scintillation fluid. The radioactive signal was measured with a PerkinElmer Life Sciences Tri-Carb 2810 TR liquid scintillation analyzer. Each reaction was performed in triplicate. The counts per minute were converted to molar units using a standard curve of known [^14^C]acetyl-CoA concentrations in the scintillation fluid.

### Peptide synthesis

For CD studies, peptides were synthesized to contain the first 7 residues (Htt_2–8_: ATLEKLM) and the first 16 residues (Htt_2–17_: ATLEKLMKAFESLKSF) of the N-terminal domain from Htt, after methionine excision. The C-termini of the corresponding peptides were amidated, and the N-terminus was either left unmodified (Htt_2–8_ and Htt_2–17_) or N-terminally acetylated (Ac-Htt_2–8_ and Ac-Htt_2–17_).

For radioactive acetyltransferase assays, all peptides were synthesized with an unmodified N-terminus and an amidated C-terminus. With the exception of the human histone H4 peptide, the peptides were synthesized with the first 7 residues of the corresponding substrate (Htt or the NatC *in vivo* substrate, *gag*) followed by a positively charged poly-arginine tag for electrostatic capture by the phosphocellulose papers used in the assays. The *in vitro* NatA substrate, histone H4 peptide, consisted of the first 19 residues of human H4.

All peptides were synthesized by Genscript. Full peptide sequences for activity assays are as follows:

Htt: NH_2_-ATLEKLMRWGRPVGRRRRP-CONH_2_

Histone H4: NH_2_-SGRGKGGKGLGKGGAKRHR- CONH_2_

*gag*: NH_2_-MLRFVTKRWGRPVGRRRRP- CONH_2_

### MALDI MS

Unmodified (∼49 μM) and N-terminally acetylated recombinant, uncleaved HttQ44-SUMO (∼33 μM) in sizing buffer were diluted 1:10 using Milli-Q water. The diluted sample was then mixed with matrix consisting of saturated sinapic acid solution in a mixture of 30% acetonitrile and 70% Milli-Q-grade water containing 0.1% trifluoroacetic acid. MALDI mass spectra were collected using a Bruker Ultraflex III MALDI-TOF-TOF mass spectrometer with a molecular weight window of 5 to 21 kDa.

### TEM of Htt aggregates

Unmodified and N-terminally acetylated HttQ44-SUMO proteins (10 μM) as well as unmodified and Ac-HttQ25 SUMO protein (8 μM) were each allowed to aggregate in parallel by incubation at RT overnight in sizing buffer without TEV cleavage in low-bind tubes. To quench the aggregation process, 5 μl of the sample was spotted onto a 300-mesh formvar carbon-coated copper grid (Electron Microscopy Sciences), washed, stained with 2% (w/v) uranyl acetate, and washed again to remove excess stain before air drying. A buffer sample was also prepared at t = 0 and a final (24-h time point) sample to monitor as a background/negative control. Samples were then visualized using a Jeol-1010 transmission electron microscope.

### CD spectroscopy

CD measurements were performed using an AVIV Circular Dichroism Spectrometer Model 410 where isothermal wavelength scans were collected at 37 °C. Quartz cuvettes having 1.0-cm path length were used for CD measurements. Htt 7mer peptides were studied using a final concentration of 200 μM (unmodified) and 245 μM (N-terminally acetylated), while Htt 16mer peptides were studied using a final concentration of 36 μM (unmodified) and 68 μM (N-terminally acetylated). All peptides were dissolved in a buffer containing DPBS, pH 7.4. Each spectrum was the average of three acquisitions recorded. The resulting spectra were all corrected using buffer scans collected under the same conditions.

### DLS

After dilution with the size-exclusion buffer, Htt particles (20.9 μM) were carefully pipetted into a quartz cuvette that had been previously equilibrated at 37 °C for 5 min. Aggregation was induced by addition of TEV protease (0.012 mg ml^−1^) and monitored without agitation at a constant temperature of 37 °C by DLS using a Zetasizer μV dynamic light scattering instrument (Malvern Instruments). Each sample was measured 120 times with 11 runs of 10 s and a delay of 30 s between measurements where the first measurement was taken 30 s after addition of TEV. Volume distribution reported corresponds to data extracted from t = 0, 0.5, 1.5, and 3 h (Fig. 2, *E*–*H*), while the mean volume was monitored over 10 h of corresponding experiments (Fig. 2*I*). Traces are representative. Samples were analyzed in triplicate (HttQ44, Ac-HttQ44, Ac-HttQ25) and duplicate (HttQ25).

### Crosslinking

Without cleavage by TEV, soluble HttQ44-SUMO and Ac-HttQ44-SUMO protein (23.7 μM; 0.48 mg ml^−1^) were incubated in the sizing buffer at RT for 30 min with increasing concentrations (0, 4.7, 9.4, 18.8, 37.5, and 75 mM) of DSS (Thermo Fisher: cat # 21655) or EGS (Thermo Fisher: cat # 21565) prepared in DMSO. While DSS has a homobifunctional NHS ester with a 11.4-Å arm spacer, EGS has a homobifunctional NHS ester with a 16.1-Å arm spacer. Reactions were quenched by addition of 1 M Tris, pH 7.5, to a final concentration of 16 mM. Reactions were then supplemented with SDS-loading buffer and boiled for 5 min, and then loaded onto a 15% SDS-PAGE gel.

Crosslinked samples were visualized by Western blot. To perform the Western blot, the protein was transferred to a PVDF membrane, blocked with 5% nonfat milk in TBST, incubated for 1 h with the primary antibody as described above, washed for 5 min five times with TBST, and then incubated for an hour at RT with IRDye 680RD Goat anti-mouse (LI-COR, cat # 926-68070) secondary antibody. After an additional set of 5-min washes (5 times), the membrane was washed with TBS for 5 min, to remove residual Tween. Images were acquired using an Odyssey CLx Imaging system with Image Studio v2.0.38 software (LI-COR Biosciences). The resulting imagines were cropped and arranged using Adobe Illustrator software v. 24.2.

### Turbidity aggregation assays

Turbidity used to monitor spontaneous fibrilization was performed in a 384 transparent flat nonbinding multi-well microscopy plate (Greiner Bio-One). To initiate aggregation, TEV protease (1:5 w/w Htt:TEV) was carefully pipetted into select wells containing the sizing buffer and 10 μM HttQ44 (6.04 μg), Ac-HttQ44 (6.04 μg), HttQ25 (10.6 μg), or Ac-HttQ2 (10.6 μg), resulting in a total volume of 30 μl. The plate was then immediately sealed with MicroAmp Optical Adhesive Film (Applied Biosystems, cat # 4311971) to minimize evaporation and spun down to remove any remaining air bubbles.

Turbidity of the solutions was monitored without agitation every minute by measuring the absorbance at 405 nm (10 flashes) with a target temperature of 37 °C using a TECAN Spark Multimode Microplate reader. Owing to the inherent variability of amyloid aggregation, data were collected as a series of 3 technical replicates. Turbidity traces presented in Figure 3*A* are representative curves with individual traces shown in Figure S4 demonstrating the variability of the assay.

Kinetic parameters were calculated in GraphPad Prism 9 using a three-parameter dose–response curve with a least-squares regression fitting method:

$$Y=Y_{i}\;+\;\frac{t\left(Y_{f}\;-\;Y_{i}\right)}{\left(t_{50}\;+\;t\right)}$$

Where *Y*_*f*_ and *Y*_*i*_ are the final and initial, respectively, plateaus of the curve, *t*_*50*_ is the aggregation half-life, and *t* is the time in hours.

## Data availability

All data are contained within the article except for the results of Edman degradation performed on purified HttQ44-SUMO. These data are available upon request from the corresponding author.

## Supporting information

This article contains supporting information.

## Conflict of interest

The authors declare that they have no conflicts of interest with the contents of this article.
