# Supplementary material for: N-alpha-acetylation of Huntingtin protein increases its propensity to aggregate
Source: J Biol Chem. 2021 Oct 31;297(6):101363. doi: 10.1016/j.jbc.2021.101363 (PMC8640455; doi:10.1016/j.jbc.2021.101363)
Supplement: Figures S1–S5 [file mmc1.pdf]

N-alpha-acetylation of Huntingtin protein increases its propensity to aggregate

Leah Gottlieb[1,2], Lin Guo[1,3], James Shorter[1] and Ronen Marmorstein[1,2]

[1] Department of Biochemistry and Biophysics, Perelman School of Medicine, University of Pennsylvania, Philadelphia, PA 19104
[2] Abramson Family Cancer Research Institute, Perelman School of Medicine, University of Pennsylvania, Philadelphia, PA 19104
[3] Current Address: Department of Biochemistry and Molecular Biology, Thomas Jefferson University, Philadelphia, PA 19104

Supporting Information

Supplementary Figures and Legends
Supplemental Experimental Procedures

Supplementary Figures and Legends

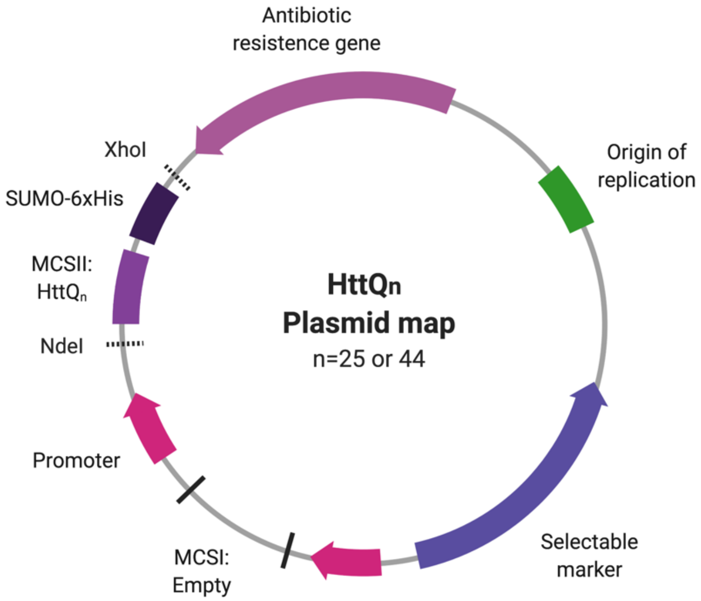

Figure S1. Htt Vector Map.
General schematic of vector map for C-terminally SUMO-6xHis-tagged HttQ25 and HttQ44 protein constructs engineered into either a pRSF or pET DUET vector used for recombinant expression in BL21-(DE3)pLysS *E. coli* cells. Figure prepared using Biorender.com

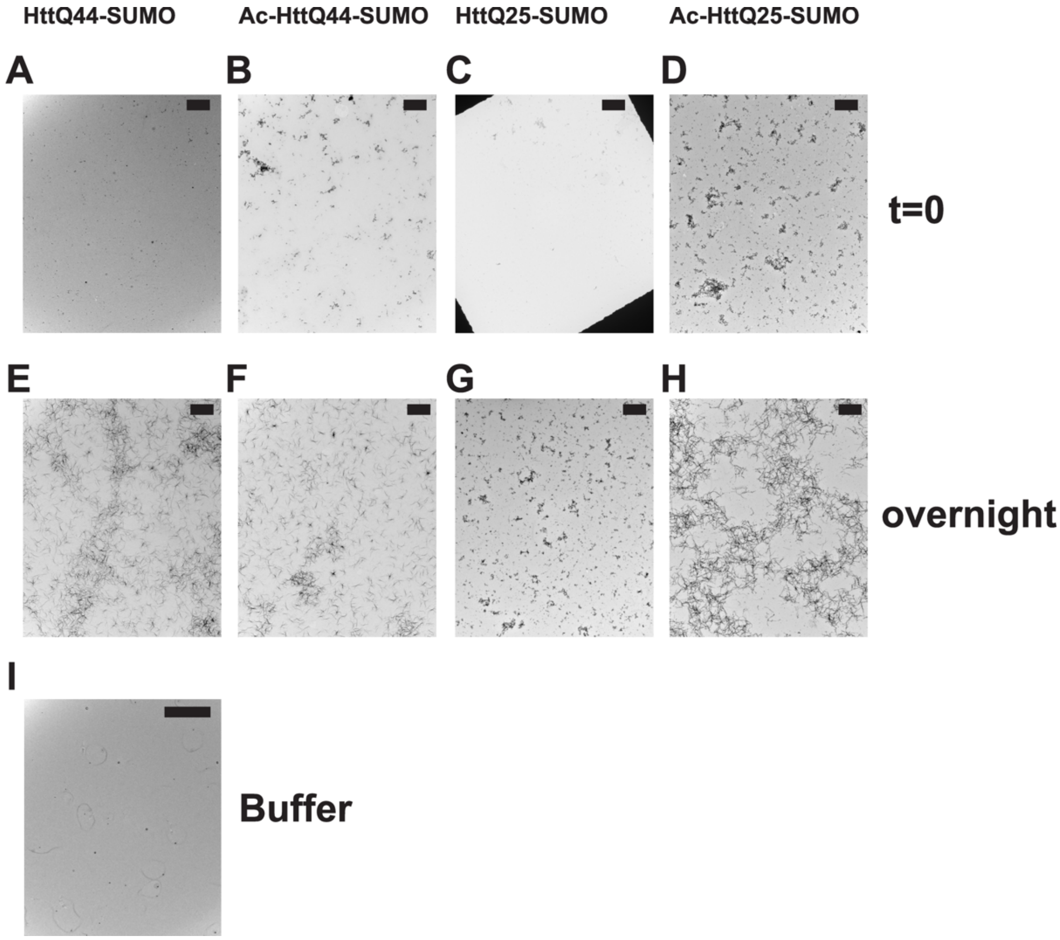

Figure S2. Low Magnification TEM Conditions
Representative low-magnification electron micrograph images containing Htt proteins (10 μM): A,E) HttQ44-SUMO; B,F) Ac-HttQ44-SUMO; C,G) HttQ25-SUMO; and D,H) Ac-HttQ25-SUMO, sampled prior to (A-D) and after (E-H) overnight incubation at ambient temperatures. I) Buffer-only micrograph sampled only after parallel overnight incubation. Scale bars are 4 μm.

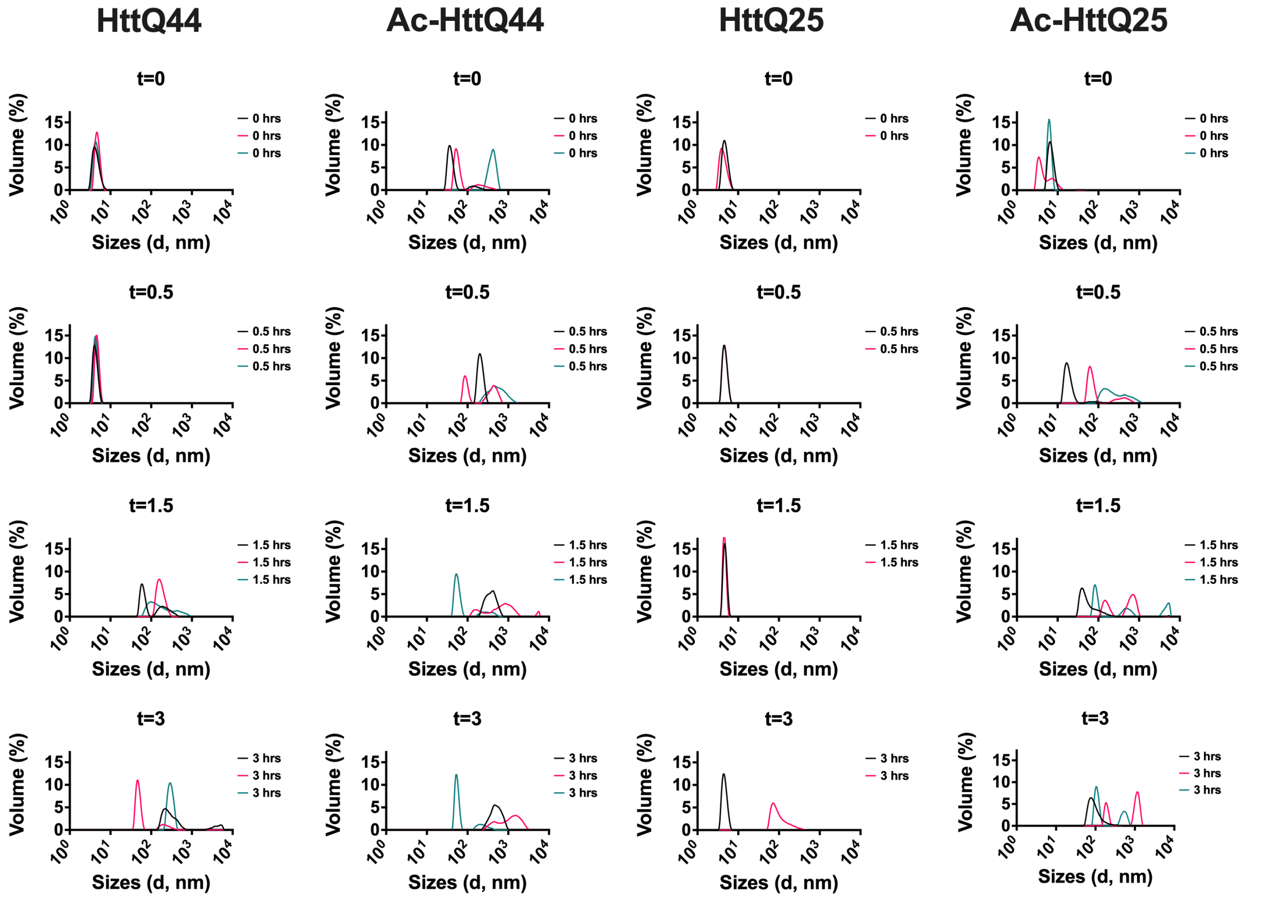

Figure S3. DLS Replicates
Replicate traces of raw DLS time course with Htt proteins (20 µM) for each time point corresponding to representative traces in Fig 2E-H. Experiments were performed as biological triplicates, except for HttQ25, which was performed in biological duplicate.

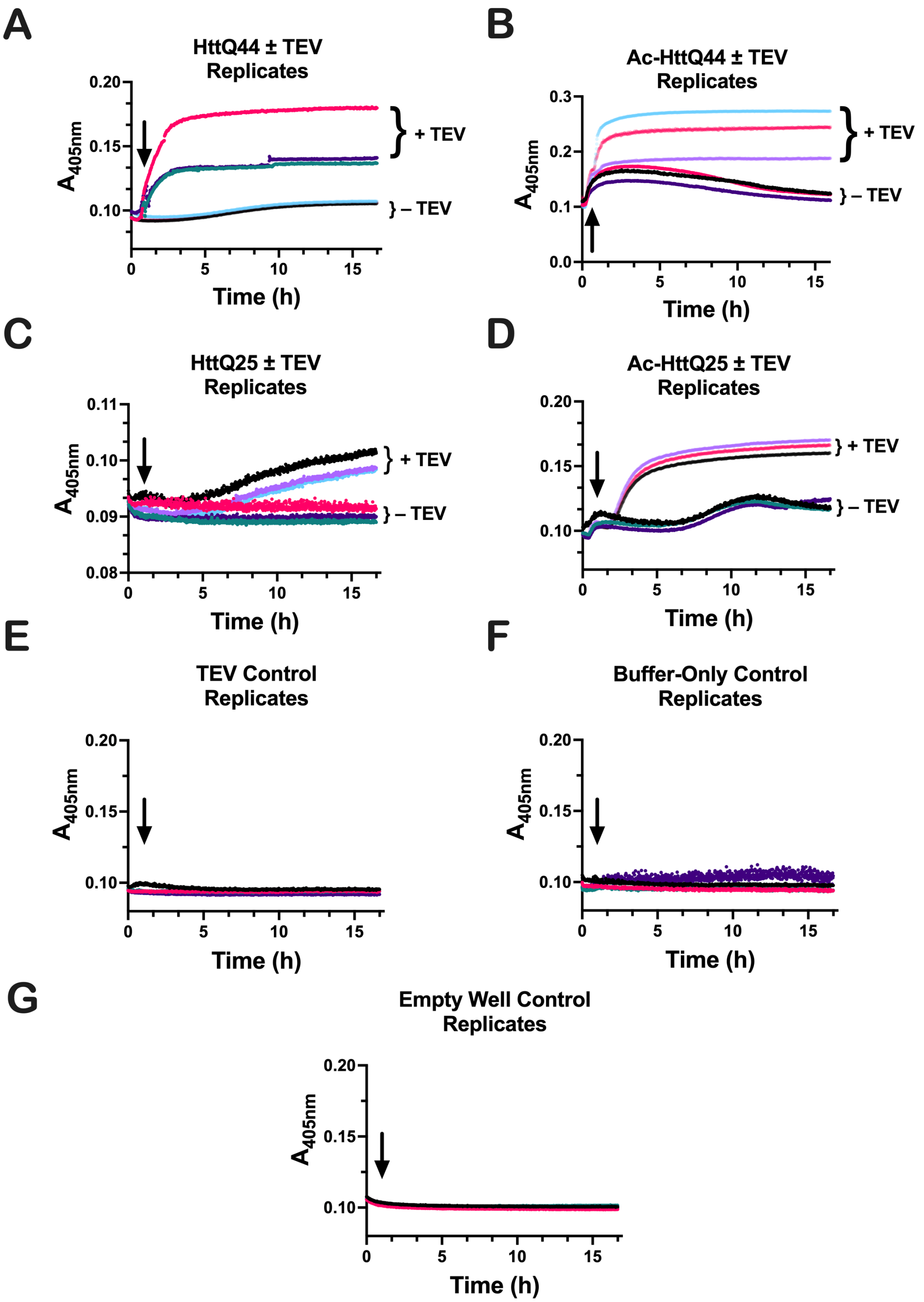

Figure S4. Turbidity assay replicates and control sample replicates. Individual raw traces of Htt protein incubated without (–) and with (+) TEV cleavage monitored at 405 nm, where t = 0 is the first reading: A) HttQ44, B) Ac-HttQ44 C) HttQ25 and D) Ac-HttQ25; and control samples E) TEV only, F) buffer only, and G) wells without buffer. Arrow indicates region where we observed a consistent anomaly across data sets.

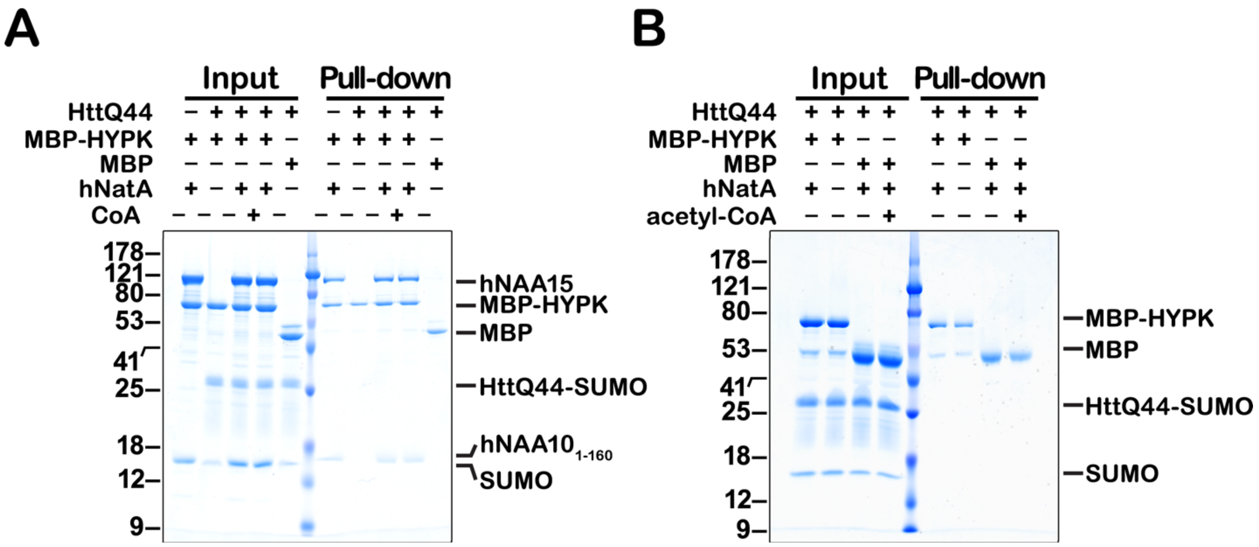

Figure S5. MBP-HYPK does not pull-down monomeric HttQ44-SUMO.
MBP pull-down assays evaluating the interaction between either MBP-HYPK or MBP (2 μM) with HttQ44-SUMO (6 μM) when A) in the presence or absence of either hNatA (2 μM) or hNatA (2 μM) and CoA (100 μM); or B) when N-terminally acetylated after overnight incubation with acetyl-CoA co-factor (100 μM) and sub-stochiometric levels of hNatA (20 nM).

Supplemental Experimental Procedures

Pull-down assays

Unmodified, uncleaved HttQ44-SUMO, 6xHis-tagged hNatA, and both free MBP as well as MBP-tagged HYPK were all prepared as described previously (1). The pull-down experiments represented in **(Figure S6)** were conducted by incubating 2 μM MBP-tagged protein (or free MBP) with 6 μM bait (6xHis-hNatA and/or unmodified HttQ44-SUMO) with 100 μM CoA in sizing buffer at 4°C for 30 min. Proteins were then subjected to pull-down by incubation with amylose agarose resin (70 μL slurry, New England BioLabs) for 30 min. Resin was washed with 80 CV of sizing buffer before elution of bound proteins by a 15-minute incubation with sizing buffer supplemented with 40 mM maltose.

In order to N-terminally acetylate Htt protein *in vitro* for pull-down analysis unmodified HttQ44-SUMO was incubated overnight in sizing buffer at 4°C (to minimize aggregation) with 100 μM acetyl-CoA and 20 nM NatA. Sub-stoichiometric amount of human NatA were used in order to maximize HttQ44-SUMO protein N-terminal acetylation while minimizing visualization of the hNatA complex by SDS-PAGE gel. Unacetylated and acetylated HttQ44-SUMO was then subjected to pull-down analysis by incubating 2 μM MBP-tagged protein (or free MBP) with 6 μM bait (unmodified or acetylated HttQ44-SUMO). The pull-down was then conducted following the same procedure outlined above.

Results of all pull-down assays were analyzed though visual inspection of input and pull-down samples with 15% SDS-PAGE. Gels were stained using Coomassie Brilliant Blue G-250.

References Cited

1. Gottlieb, L., and Marmorstein, R. (2018) Structure of Human NatA and Its Regulation by the Huntingtin Interacting Protein HYPK. *Structure* **26**, 925-935 e928
